# Effect of Zinc Priming on Salt Response of Wheat Seedlings: Relieving or Worsening?

**DOI:** 10.3390/plants9111514

**Published:** 2020-11-08

**Authors:** Carmelina Spanò, Stefania Bottega, Lorenza Bellani, Simonetta Muccifora, Carlo Sorce, Monica Ruffini Castiglione

**Affiliations:** 1 Department of Biology, University of Pisa, 56126 Pisa, Italy; stefania.bottega@unipi.it (S.B.); carlo.sorce@unipi.it (C.S.); monica.ruffini.castiglione@unipi.it (M.R.C.); 2 Centre for Climate Change Impact, University of Pisa, 56124 Pisa, Italy; 3 Department of Life Sciences, University of Siena, 53100 Siena, Italy; lorenza.bellani@unisi.it (L.B.); simonetta.muccifora@unisi.it (S.M.); 4 Institute ofAgricultural Biology and Biotechnology (IBBA), National Research Council, 56124 Pisa, Italy

**Keywords:** bulk zinc oxide, foliar spray, root zone treatment, *Triticum turgidum*, zinc oxide nanoparticles

## Abstract

In an attempt to alleviate salt-induced damage, the application of ZnO nanoparticles has been suggested. As the use of these particles has also been associated with phytotoxicity, to better clarify the effect of zinc and its possible mitigation of salt stress, we treated wheat seedlings with ZnO (nanoparticles or their bulk-scale counterparts, amended either in the growth medium, NPs and B, or sprayed on the leaves, SPNPs and SPB) with or without subsequent treatment with salt. Growth, photosynthetic parameters, zinc and ion concentration, and in situ and biochemical determination of oxidative stress in wheat leaves and/or in roots were considered. Both Zn and NaCl significantly inhibited growth and induced severe alterations in root morphology. Oxidative stress and damage decreased or increased under ZnO treatment and in saline conditions depending on the organ and on the size and mode of application of particles. In spite of the higher stress conditions often recorded in treated leaves, neither pigment concentration nor photochemical efficiency were decreased. A large variability in the effects of ZnO treatment/priming on seedling salt response was recorded; however, the presence of a cumulative negative effect of priming and salt stress sometimes observed calls for caution in the use of ZnO in protection from saline stress.

## 1. Introduction

Soil salinity, affecting at least 20% of cultivated lands, is one of the main problems for crop productivity as salt can impair growth and development of plants. Salt, besides inducing osmotic stress, can cause toxicity elicited by the high uptake of ions such as Na^+^ and Cl^−^. Nutritional imbalance and generation of reactive oxygen species (ROS) with resulting oxidative stress have also been reported [1]. As a consequence of these disturbances, several physiological processes, such as photosynthesis and water balance, can be impaired [2]. The application of nutrients can mitigate the deleterious effects of salinity, and in particular, the protective action of zinc has been reported [2,3,4]. Zinc is a micronutrient necessary for the optimal growth and development of plants. In fact, according to literature, zinc, besides affecting plant water status, is necessary for the synthesis of indole-3-acetic acid (IAA) and for membrane integrity and enzymatic activities, and participates in synthesis and stability of nucleic acids and in metabolism of carbohydrates and lipids (see [5] and the references therein). Zinc application, besides having positive effects on physiological parameters [6], can mitigate the negative effects induced by abiotic stresses in plants, with improvement of plant performance in the presence of heavy metal contamination [7,8], in drought conditions [9], and under salt treatment [4,10]. ZnO nanoparticles (NPs), being more reactive than their bulk-scale counterparts mainly for their high surface area, have been suggested as useful to improve plant salt tolerance [3,11]. Foliar application of NPs, in particular, is generally considered an eco-friendly practice of micronutrient fertilization, as foliar amendment could reduce the toxicity symptoms linked to soil application [12]. In fact, in the literature, besides the abovementioned positive effects, there are also data reporting that ZnO NPs impair plant growth [13], enhance reactive oxygen species (ROS) generation [14] lignification and cell death, thereby probably inducing the inhibition of root growth in seedlings [15], and have a negative impact on several species (see [16] and the references therein). Nanoparticles are able to overcome the first barriers of plant cell, mainly exploiting pores in the cell wall and the endocytosis pathways until they can reach the vascular system and the whole plant body [17]. With regards to ZnO NPs, the related phytotoxicity can derive from the release of Zn^2+^ [18], from the direct interaction of ZnO NPs with biomolecules with damage to membranes and/or DNA [19], and from the ZnO NP-induced generation of ROS, such as hydrogen peroxide (H_2_O_2_) [20]. The overproduction of ROS can induce membrane lipid peroxidation and cellular damage, considered as one of the primary contributors to nanotoxicity in plants [17]. As ZnO NPs have a wide range of applications [21] and their intentional or unintentional release into the environment could result in increasing contamination, the knowledge of the real impact of these particles on living organisms, in particular plants, is mandatory.

Wheat is considered a staple food for about 50% of the worldwide population. Its salt tolerance differs among different cultivars and durum wheat is usually considered a moderately salt tolerant species [22,23]. Despite the phytotoxicity associated with ZnO NPs, data in the literature report that zinc application can alleviate damages induced to plant under saline conditions [10]. In an attempt to clarify the effect of zinc on salt tolerance, in the present research, we treated *Triticum turgidum* ssp. *durum* cv. Cappelli seedlings with salt after priming with ZnO. Both NPs and their bulk counterparts were used for the priming treatment to highlight possible differences linked to the size of particles. Due to the peculiar advantages of foliar application of nutrients, ZnO (NPs and bulk) was amended either in the growth medium or sprayed on the leaves. Growth, photosynthetic parameters, zinc and ion concentration, and in situ and biochemical determination of oxidative stress were measured in wheat leaves and or in roots. Our aims were (i) to assess the effect of pretreatment with ZnO on growth and physiological parameters in wheat, highlighting eventual size- and mode-of-application-dependent differences and (ii) to evaluate the role of ZnO priming in the modulation of wheat response to salt.

## 2. Results and Discussion

### 2.1. ZnO NPs and Bulk Characteristics 

**Figure 1:**
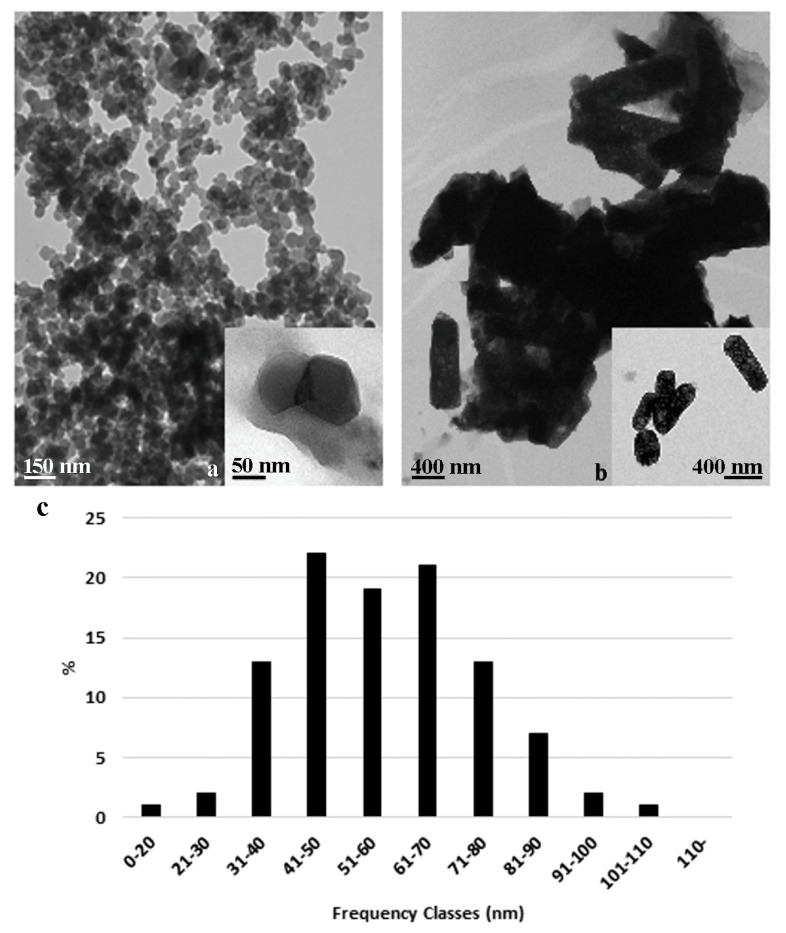
Transmission electron micrograph of the nanoparticle (**a**) and bulk form (**b**) of ZnO added in the root zone or as a leaf spray: size distribution of nanoparticles grouped in frequency classes (**c**) after an ImageJ program elaboration of TEM images.

Under TEM, both the nano and the bulk form of ZnO particles (Figure 1a,b) appeared highly aggregated. The ZnO NPs (Figure 1a) showed a round profiled or slightly prismatic shape with little difference in the length of axes, so as to consider them almost spherical, the diameters varying from about 20 nm to 107 nm (Figure 1a). Most of the NPs had diameters between 41–50 nm and 61–70 nm (Figure 1c), and about 3% showed diameters less than 30 nm and more than 90 nm (Figure 1c). The bulk particles had predominantly a rod-like shape and sizes varying from 300 nm to 1300 nm (Figure 1b). Particles with one dimension smaller than 80–90 nm have been seldomly observed in the B sample.

### 2.2. Zinc Concentration

**Figure 2:**
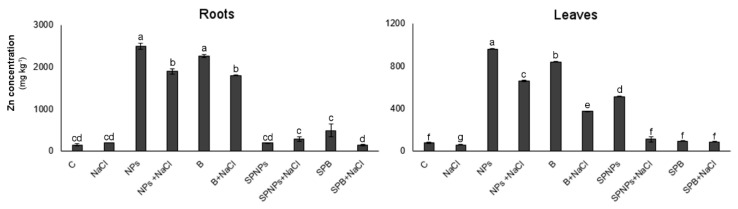
Zinc concentration in roots and leaves of *T. turgidum* L. ssp. *durum* cv. Cappelli without priming and salt treatment (C), with salt treatment without priming (NaCl), with ZnO priming in the root zone without salt treatment (nanoparticles, NPs or bulk form, B), with ZnO priming in the root zone with salt treatment (nanoparticles, NPs + NaCl or bulk form, B + NaCl), with ZnO priming as a leaf spray without salt treatment (nanoparticles, SPNPs or bulk form, SPB), and with ZnO priming as a leaf spray with salt treatment (nanoparticles, SPNPs + NaCl or bulk form, SPB + NaCl): values are means of at least three replicates ± SE. When ANOVA highlighted significant differences, Tukey’s post hoc test was performed, and different letters indicate significantly different mean values at *p* < 0.05.

In roots, zinc concentration (Figure 2) depended on ZnO treatment and salt presence, with a significant interaction between the two factors (Table S1). The concentration of this metal significantly increased when it was added to the growth medium both as nano and as bulk ZnO. Data in the literature [24] underline the importance of particle dimensions in the uptake of this metal, but though Zn concentration was lower under B than under NPs treatment, differences were not significant. The subsequent addition of NaCl significantly reduced Zn content. Since it has long been known that Zn accumulates in the root intercellular free space [25], we can make the hypothesis that, under saline conditions, Na^+^ could replace Zn in the interaction with negative charges of the cell wall. A similar pathway was recorded in leaves (Figure 2), with Zn concentrations much lower than those detected in roots and a significant interaction between the two independent factors (Table S2). Foliar application did not increase the concentration of this element in roots but significantly increased Zn concentration in leaves when SPNPs were applied. This increase, also recorded in *Triticum aestivum* sprayed with comparable concentrations of ZnO NPs [7], was not detected when the B form was sprayed, underlining the different ability of ZnO to permeate plant tissues depending on the size of particles. The subsequent application of salt significantly reduced Zn concentration, in accordance with Khoshgoftarmanesh et al. [26], reporting a decrease in Zn concentration at increasing NaCl concentration in shoots of five different wheat genotypes. Despite this decrease, Zn concentration values were still significantly higher than those of non-primed plants. Significant was the inhibitory effect of salt on leaf Zn absorption, as evidenced by the comparison between control and NaCl-treated plants, in accordance with previous studies [27,28]. 

### 2.3. Ion Concentration

The Na^+^ root concentration changed with significant impact from the two independent factors also in interaction (Tables S1 and S2). As expected, the concentration of Na^+^ was significantly increased in comparison with the control when plants were treated with NaCl (Table 1). The priming treatment with bulk ZnO, both in the medium and as a spray, and SPNPs before salt application significantly reduced this concentration in roots (Table 1). In leaves of NPs + NaCl and SPB + NaCl, a decrease in the concentration of Na^+^ in comparison with NaCl was recorded (Table 1). Our data are in accordance with the already reported inhibition of Na uptake and translocation induced by Zn [29]. The trend of Cl^-^ ion concentration was the same as that recorded for Na^+^ (Table 1).

In all treatments, a significant increase in root K^+^ concentration in comparison with control plants was recorded, with a significant effect of ZnO treatment and salt presence and of the interaction between them(Table S1). Differently from most literature data [30,31,32], this increase also involved salt-treated roots (Table 1). In accordance, some studies reported an increase in root K^+^ content in saline conditions (see [33] and the references there in) that, together with the high Ca^2 +^ concentration (Figure S1), could help to maintain a good water status in NaCl-treated roots [34], decreasing the need for the expensive synthesis of organic osmolites. ZnO pretreatment further increased K^+^ concentration in the saline condition, in particular, in roots. The recorded increase in K^+^ concentration did not correspond to an increase in K^+^/Na^+^ ratio (Table 1), due mainly to the striking increase in Na^+^ concentration. In fact, salt treatment always significantly reduced K^+^/Na^+^ ratio, and although B and SPB significantly increased this ratio, the subsequent treatment with NaCl significantly reduced it to values lower than those of control plants. As an adequate K^+^/Na^+^ ratio is considered important for salt tolerance, Zn priming did not seem to improve the performance of wheat seedlings under our experimental conditions.

### 2.4. Plant Growth and Root Morphology 

**Figure 3:**
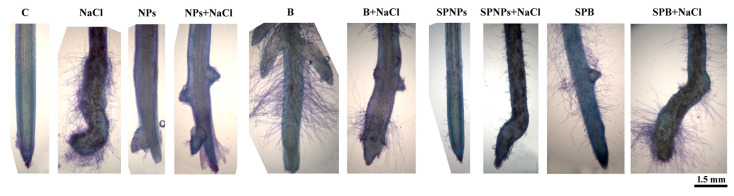
Roots of *T. turgidum* L. ssp. *durum* cv. Cappelli stained with toluidine blue and observed using an optical microscope to evaluate their general morphology and architecture: roots without priming and salt treatment (C), with salt treatment without priming (NaCl), with ZnO treatment in the root zone (nanoparticles, NPs or bulk form, B), with ZnO priming in the root zone with salt treatment (NPs + NaCl or B + NaCl), with ZnO treatment as a leaf spray (SPNPs or SPB), and with ZnO priming as a leaf spray with salt treatment (SPNPs + NaCl or SPB + NaCl).

Treatments with NPs and B caused an altered root morphology in wheat (Figure 3), characterized by a more acropetal induction of lateral root primordia in comparison with control: very close to the apical root meristem in NPs and more distant from the apex in B plants (Figure 3). In addition, damage/shortening of the root tip was recorded, as well documented following ZnO NP treatments in ryegrass [18]. This wide Zn-dependent remodelling of root morphology has been previously described in wheat [35,36] and in other plant species, as for example in *Brassica* [37], and could be derived from the ability of Zn to act on hormonal homeostasis, especially of auxin, that together with cytokinins and ethylene are able to regulate lateral root initiation [38]. In accordance, as speculated by Cadiz and Davies [39] in grass species, the increase in branching detected under Zn treatment might be the result of partial loss of the dominance of the damaged root apex that triggers changes in hormonal balance/transport. A comparable but less accentuated morphogenic response was found in the corresponding spray treatments (Figure 3), suggesting the ability of ZnO to exert long-distance effects from leaves to the root apparatus. In B plants, root hair emergence occurred at a shorter distance from the apex as an outstanding thick trichomatous complex (Figure 3). This acropetal shift of root hair development, the effect in our system of a Zn-dependent induced morphogenic response, was found in other plant species under As and Cd treatments [40,41]. Salt treatment extremely reduced the size of the meristematic zone of the root (Figure 3), as reported for *Arabidopsis* [42], with a consequent effective acropetal shift of root hair cell development. In accordance with Munns [43], the recorded increase in root diameter (1.35 ± 0.21 mm under saline conditions and 0.89 ± 0.063 mm for control roots, with a 50% increase, Figure 3) may be an anatomical adaptation to store more water in the vacuolar compartment of the cortex cells. The observed browning of epidermis cell walls, possibly due to impregnation by protective phenolic compounds (Figure 3), is a typical trait induced by stressors [44] and can be considered another adaptive response. The anomalous morphology characterized by induction of root branching, associated with Zn treatment, persisted also after salt addition.

**Figure 4:**
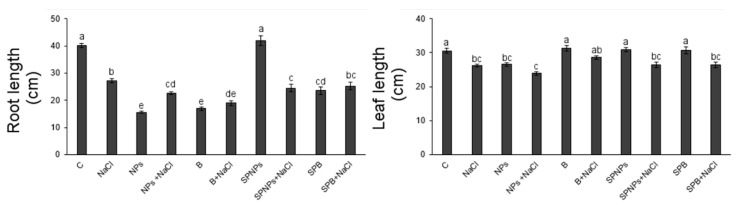
Length of roots and leaves of *T. turgidum* L. ssp. *durum* cv. Cappelli without priming and salt treatment (C), with salt treatment without priming (NaCl), with ZnO treatment in the root zone (nanoparticles, NPs or bulk form, B), with ZnO priming in the root zone with salt treatment (NPs + NaCl or B + NaCl), with ZnO treatment as a leaf spray (SPNPs or SPB), and with ZnO priming as a leaf spray with salt treatment (SPNPs + NaCl or SPB + NaCl): values are means of at least three replicates ± SE. When ANOVA highlighted significant differences, Tukey’s post hoc test was performed, and different letters indicate significantly different mean values at *p* < 0.05.

Significant was the impact on root growth of the two factors also in the interaction (Table S1). All treatments with the exception of SPNPs significantly reduced root length in comparison with control plants (Figure 4). Both salt application and treatment with ZnO supplied in the growth medium caused a significant reduction in the length of this organ (about 32% and 61%, respectively) partially compensated by an increase in root diameter and branching, respectively. Noteworthily, there was a partial recovery in plants treated with salt after ZnO pretreatment (43%). Significant was the influence of ZnO treatment and salt on leaf growth (Table S2). Leaf length (Figure 4) was always reduced in plants treated with NaCl (14%), in particular, in plants pretreated with NP ZnO in growth medium where the effect of NaCl amplified the inhibiting effect of NPs (about 22% of inhibition). Noteworthily, in NaCl plants, the reduction in growth was associated with an overaccumulation of Na^+^ plus K^+^ (about 26,000 and 50,000 mg kg^−1^ in roots and leaves, respectively), as reported for *Arabidopsis* plants [1]. It is interesting to note that the reduction in growth in itself can also have a positive connotation, since it can be considered an adaptive response, saving resources available for the synthesis of protective substances [31]. 

**Figure 5:**
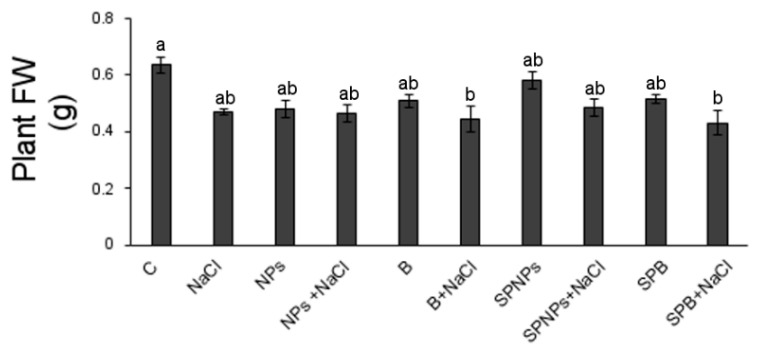
Fresh weight of plants of *T. turgidum* L. ssp. *durum* cv. Cappelli without priming and salt treatment (C), with salt treatment without priming (NaCl), with ZnO treatment in the root zone (nanoparticles, NPs or bulk form, B), with ZnO priming in the root zone with salt treatment (NPs + NaCl or B + NaCl), with ZnO treatment as a leaf spray (SPNPs or SPB), and with ZnO priming as a leaf spray with salt treatment (SPNPs + NaCl or SPB + NaCl): values are means of at least three replicates ± SE. When ANOVA highlighted significant differences, Tukey’s post hoc test was performed, and different letters indicate significantly different mean values at *p* < 0.05.

Leaf number was significantly lower in treated than in control plants, with the exception of NPs, SPNPs, and B plants (Table 2). The reduction in leaf number in salt conditions evidenced the importance of this factor for this trait (Table S2) and is in accordance with Qados [45], who reports a NaCl concentration-dependent decrease in the number of leaves in *Vicia faba*. Fresh weight (FW, Figure 5) was only slightly reduced in treated plants and NPs, known from literature to be able to reduce plant weight [46], reduced this parameter in a not significant manner. Only in B + NaCl and SPB + NaCl plants, the reduction, in comparison with C plants, was significant and the minimum growth was recorded. The negative effect of salt on plant growth is commonly reported in the literature [45,47]; however, contrasting data characterize plant growth response to ZnO [7,16,46,48], and Zn phytotoxicity has been observed depending, among the others, on plant species and age [5].

### 2.5. Relative Water Content, Pigments Concentration, and Chlorophyll Fluorescence

**Figure 6:**
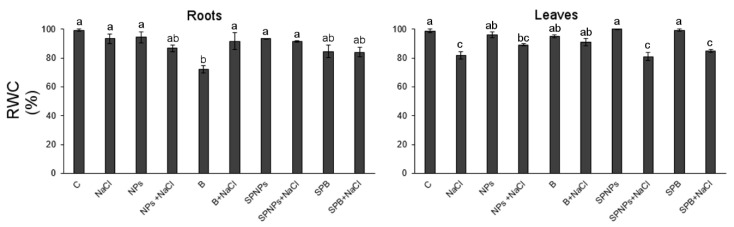
Relative water content (RWC) of roots and leaves of *T. turgidum* L. ssp. *durum* cv. Cappelli without priming and salt treatment (C), with salt treatment without priming (NaCl), with ZnO treatment in the root zone (nanoparticles, NPs or bulk form, B), with ZnO priming in the root zone with salt treatment (NPs + NaCl or B + NaCl), with ZnO treatment as a leaf spray (SPNPs or SPB), and with ZnO priming as a leaf spray with salt treatment (SPNPs + NaCl or SPB + NaCl): values are means of at least three replicates ± SE. When ANOVA highlighted significant differences, Tukey’s post hoc test was performed, and different letters indicate significantly different mean values at *p* < 0.05.

In roots, the lowest value of relative water content (RWC) characterized plants treated with B ZnO in the growth medium (Figure 6). The addition of NaCl enhanced RWC until values were not significantly different from control plants. In leaves, salt was an important factor in the control of RWC (Table S2), and in accordance with previous data [23,49], it significantly decreased RWC. Water balance was better in plants pretreated with B in the growth medium but not when ZnO was amended as spray (Figure 6).

Despite the general growth inhibition recorded, neither total chlorophyll nor carotenoid concentrations changed in treated plants in comparison with control ones (Table 2), in accordance with Wang et al. [21], who found similar results in tomato treated with 200 mg L^−1^ ZnO NPs. ZnO treatment had a significant impact on chla/chlb ratio (Table S2) that showed a decrease associated with ZnO treatments, with preferential investment in the antenna by these plants (Table 2). Maximum PSII quantum yield (*Fv/Fm*) was not negatively affected by any treatment (Table 2). The sensitivity of this parameter to NaCl may change between species and within species, according to Kalaji et al. [50]: these authors showed that, in response to 120 mM NaCl, *Fv/Fm* decreased only in one cultivar of barley out of the two analysed. Moreover, the amount of Zn administered to plants might have been below the threshold needed for lowering *Fv/Fm*: Redondo-Gomez et al. [51] demonstrated that 1 mM Zn had no effect on *Fv/Fm* in *Spartina densiflora*. Photochemistry seemed to be unaffected under all experimental conditions, as suggested also by the stability of the concentration of photosynthetic pigments.

### 2.6. Oxidative Stress

**Figure 7:**
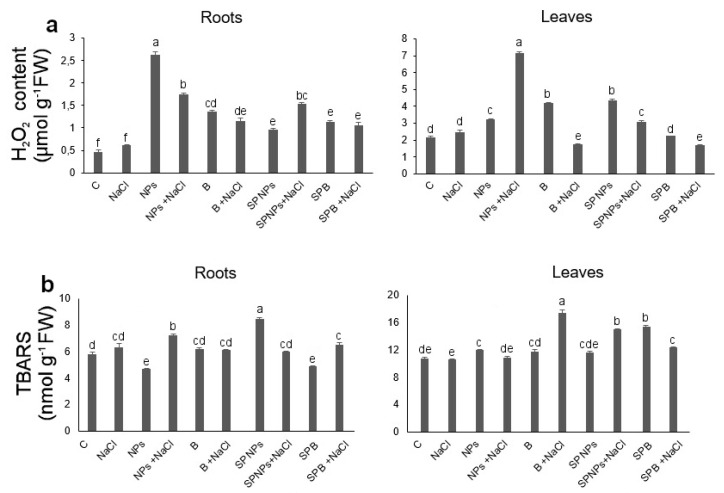
Hydrogen peroxide concentration (**a**) and thiobarbituric acid reactive substances (TBARS) concentration (**b**) in roots and leaves of *T. turgidum* L. ssp. *durum* cv. Cappelli without priming and salt treatment (C), with salt treatment without priming (NaCl), with ZnO treatment in the root zone (nanoparticles, NPs or bulk form, B), with ZnO priming in the root zone with salt treatment (NPs + NaCl or B + NaCl), with ZnO treatment as a leaf spray (SPNPs or SPB), and with ZnO priming as a leaf spray with salt treatment (SPNPs + NaCl or SPB + NaCl): values are means of at least four replicates ± SE. When ANOVA highlighted significant differences, Tukey’s post hoc test was performed, and different letters indicate significantly different mean values at *p* < 0.05.

**Figure 8:**
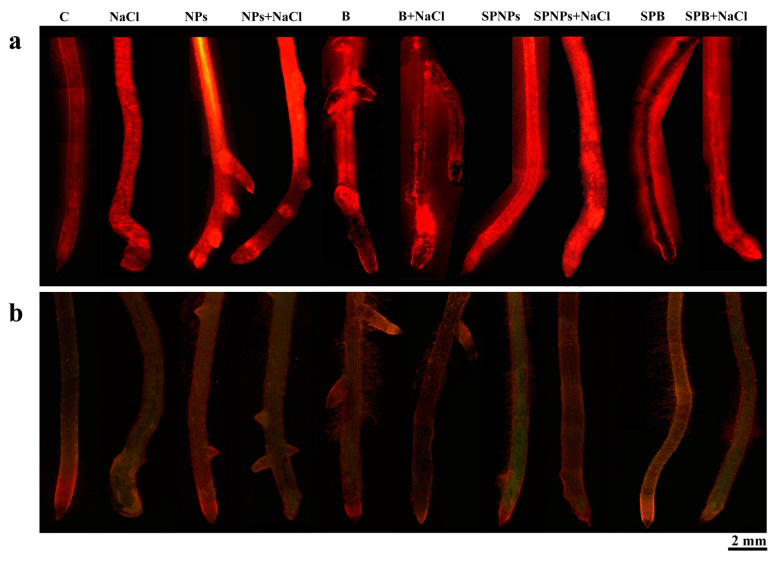
Histochemical analysis of root of comparable developmental level, randomly selected from plants without priming and salt treatment (C), with salt treatment without priming (NaCl), with ZnO treatment in the root zone (nanoparticles, NPs or bulk form, B), with ZnO priming in the root zone with salt treatment (NPs + NaCl or B + NaCl), with ZnO treatment as a leaf spray (SPNPs or SPB), and with ZnO priming as a leaf spray with salt treatment (SPNPs + NaCl or SPB + NaCl): the plate comprehends representative images of in situ detection of H_2_O_2_ by the Amplex UltraRed Reagent (**a**) and in situ detection of TBARS by the BODIPY reagent (**b**).

Though data in the literature report the induction of oxidative stress in wheat under saline conditions [23], H_2_O_2_ concentration did not significantly increase in salt-treated plants in comparison with C plants (Figure 7a), the latter being however the least reactive to the treatment with Amplex Ultrared probe staining (Figure 8a). In roots, the concentration of this signalling molecule varied in function of ZnO treatment and salt presence, also in interaction (Table S1). Though H_2_O_2_ concentration was generally significantly higher in treated plants than in C plants, the presence of NaCl after pretreatment with ZnO in the growth medium (NPs + NaCl and B + NaCl) decreased the concentration of H_2_O_2_ in comparison with plants subjected to the sole ZnO treatment (Figure 7a). The addition of NaCl also affected the staining pattern obtained with in situ H_2_O_2_ localization (Figure 8a), with a weaker response of the last part of the root in NPs + NaCl versus NPs and with an opposite tendency in B + NaCl versus B treatment. This fact suggests that ZnO can act differently depending on its form in accumulating/distributing H_2_O_2_ following salt amendment. In plants treated with NaCl after spray priming with NPs, the concentration of this signalling molecule was higher than in SPNPs plants (Figure 7a), as also recorded in histochemical analysis (Figure 8a). Though there were no significant differences in H_2_O_2_ content between the roots of SPB and SPB + NaCl plants, the red signal of Amplex Ultrared probe, mainly confined to the tegumental tissues in SPB, spread to the whole root in SPB + NaCl plants. These results highlight the complementarity of histochemical and biochemical analyses. The highest concentration of H_2_O_2_ was recorded in NPs plants, in which a significant increase in the concentration of this signalling molecule was detected (Figure 7a), in accordance with previous studies clearly indicating that ZnO NPs can induce oxidative stress in plants of *Triticum aestivum* [16]. Histochemical analysis (Figure 8a) confirmed biochemical data, with a strong signal involving also lateral roots. Interestingly, just in these roots, the highest concentration of Zn was also detected. In leaves, the concentration of H_2_O_2_ mainly depended on ZnO treatment (Table S2) and the highest value of this parameter was recorded in NPs + NaCl plants (Figure 7a). In B + NaCl and SPB + NaCl H_2_O_2_, the concentrations were lower than in NPs + NaCl and SPNPs + NaCl, respectively. Noteworthily, just in B treatments, Zn concentration was significantly lower than the corresponding NP-treated plants, underlining the importance of particle dimensions. Both in roots and in leaves, the two independent factors had a significant impact on thiobarbituric acid reactive substances (TBARS) concentration (Tables S1 and S2, respectively) (Figure 7), indicative of oxidative damage. This concentration was only slightly increased by salt, and in accordance with Xiao et al. [48], the treatment with ZnO NPs in the root zone induced a decrease in the concentration of these substances. Comparable results were obtained following a histochemical approach for in situ lipid peroxidation detection on the root system (Figure 8b). The amendment of NaCl after this priming treatment, however, increased damage to values higher than those recorded in salt-stressed plants without any pretreatment (Figure 7b). Neither bulk ZnO in the medium nor the subsequent salt amendment induced significant differences in TBARS concentration in comparison with the control (Figure 7b). Stress damage reached the highest level when NPs were sprayed (SPNPs, Figure 7b). Subsequent addition of NaCl significantly decreased oxidative damage. The opposite trend was recorded with SPB. A similar effect was also appreciable following BODIPY staining (Figure 8b), in which the SPNPs sample showed a more intense green dye compared to SPNPs + NaCl as well as to all the other treatments. However, in general, lipid peroxidation did not show particular specific patterns along root lengthwise differentiation, at least in the portion analysed, with the exception of constant oxidative damage at the root apical meristem when salt was present in the culture medium. This effect on root apex confirms the high sensitivity of meristem cells to salinity [52] that priming treatments are not able to mitigate, with a consequent disturbance in root meristem activity and root elongation. This oxidative damage does not seem ascribable to a local increase of H_2_O_2_ (Figure 8a) but probably could be triggered by enzyme activities coupled to salinity stress, such as lipoxigenases [53].

In leaves (Figure 7b), salt treatment did not increase TBARS concentration in comparison with control plants, and regarding ZnO treatments in the root zone, only NPs significantly increased oxidative damage. Subsequent treatment with NaCl caused both decrease (NPs + NaCl and SPB + NaCl) and increase (B + NaCl and SPNPs + NaCl) in oxidative damage. It should nevertheless be taken into account that previous studies showed that the same spray treatment was stressful for plants [23].

## 3. Materials and Methods 

### 3.1. ZnO Nanoparticles and Bulk Characterization 

Morphology and size of both the NPs and of the bulk form of ZnO were characterized by Transmission Electron Microscope (TEM, FEI Technai), by placing a drop (10 μL) of 60 mg L^−1^ suspension on grids covered by formvar and were allowed to settle, dried, and observed at 100 kv. TEM images were examined by the ImageJ programme (Image J 1.52a, National Institutes of Health, USA, http://imagej.nih.gov/ij) to analyse particles dimensions by measuring the diameters of at least 100 particles.

### 3.2. Plant Material and Experimental Design

Fully viable caryopses of *T. turgidum* L. ssp. *durum* (Desf.) cv. Cappelli (11% moisture content, 100% germination after 48 h of imbibition) were surface sterilized for 3 min in NaOCl (1%, *v*/*v*, available chlorine) and rinsed before use. Wheat grains were germinated in Petri dishes (15 replicates each of 100 grains) on water-moistened Whatman No. 2 filter paper at 23 ± 1 °C in the dark for 72 h. Plants were randomly divided into ten different treatment groups (100 plants each) and transplanted into 4 L polyethylene pots (50 plants/pot) filled with deionised water (23 °C, 12/12 h day/night photoperiod, photosynthetically active radiation, PAR, of 400 µmol m^−2^s^−1^, relative humidity of 70%). After six days, deionized water was substituted by ¼× Hoagland solution (Sigma), and after 4 more days (10 days after seed imbibition), a two-day priming treatment with 0.74 mM ZnO (in the root zone or as a leaf spray) started. After priming, 150 mM NaCl was added to the growth medium. To avoid osmotic shock, salt concentration was gradually increased (50 mM NaCl per day, until 150 mM). All solutions were continuously aerated. For priming with ZnO, NPs or the bulk (B) form were used. The experimental design is reported in Figure S2. In particular, the treatments were control (C) without priming and salt treatment, saline control (NaCl) with salt treatment without priming, ZnO priming in the root zone (nanoparticles, NPs or bulk form, B) without salt treatment, ZnO priming in the root zone with salt treatment (nanoparticles, NPs + NaCl or bulk form, B + NaCl), ZnO priming as a leaf spray (nanoparticles, SPNPs or bulk form, SPB) without salt treatment, and ZnO priming as a leaf spray with salt treatment (nanoparticles, SPNPs + NaCl or bulk form, SPB + NaCl). Spray treatments were applied once a day using a nebulization system. Spray treatments were made to avoid any contamination of the growth medium. Nineteen days after seed imbibition, seedlings were collected, washed, and immediately used for growth determination and histochemical analyses or fixed in liquid nitrogen and stored at −80 °C until use for all other analyses.

### 3.3. Atomic Absorption Spectrometry Analysis for Zinc Concentration Determination

Zinc concentration in roots and leaves was determined according to Jorhem [54] with minor modifications as in Spanò et al. [55]. In particular, the samples, after drying, were ashed in a muffle furnace at 525 °C for 2 h and ground in a porcelain mortar. The residue was digested with 65% HNO_3_ and 1 N HCl (3:1 *v/v*) and heated at 145 °C until white fumes start disappearing. The solution was filtered through a filter paper and brought up to a volume of 50 mL with bi-distilled water. A blank digest was carried out in the same way. Zinc concentration was measured in a flame atomic absorption spectrometer (Thermo Scientific, ICE 3000 series, Waltham, MA, USA).

### 3.4. Ion Concentration

After desiccation, leaves and roots were ground and extracted in distilled water. Extracts were filtered, and soluble ions were analysed using an ionic chromatograph (Dionex Dx 120, Sunnyvale, CA, USA) [56,57]. The concentration of ions was expressed as mg kg^−1^.

### 3.5. Seedling Growth, Root Morphology, and Relative Water Content

After collection, both leaf and root lengths and the number of leaves were recorded. Five roots of equal growth stage, randomly selected from control and treated plants, were isolated, stained with toluidine blue (0.5% *w*/*v* in 2.5% *w*/*v* Na_2_CO_3_), and observed using an optical microscope to evaluate their general morphology and architecture. Analyses were performed on the apical 1.5 cm segment of the root using a Leiz DMRB microscope equipped with a Leica DFC420 digital camera.

Fresh weight (*FW*) and dry weight (*DW*) were determined before and after oven drying of samples (70 °C till constant weight), respectively. Water content percentage was estimated on the fresh weight basis, and leaf relative water content, *RWC*, was determined as in Balestri et al. [58] and calculated using Equation (1):

(1) $$RWC=\frac{\left(FW-DW\right)}{\left(TW-DW\right)}\times 100$$

### 3.6. Pigment Concentration and Photosynthetic Efficiency

Leaf chlorophylls (a, b, and total) and carotenoids were extracted and determined as in Spanò and Bottega [23]. The concentrations of pigments were expressed as mg g^−1^FW. Nineteen days after imbibition, before the collection of seedlings, photosynthetic efficiency was determined by analysing chlorophyll a fluorescence by a portable fluorometer (MINI-PAM Walz, Effeltrich, Germany), according to Sorce et al. [59]. Eight records per pot, each one on a distinct plant, were taken on leaves dark adapted for 30 min by dark leaf clips (Walz) before the measurement of Fo, Fm, and *Fv/Fm*; the latter is an expression of the maximum PSII quantum yield [60]. Consequently, each time, the value of each thesis was the average of 8 measurements ± SE.

### 3.7. Hydrogen Peroxide and TBARS Determination

The hydrogen peroxide concentration of roots and leaves was determined according to Jana and Choudhuri [61]. Samples were ground and homogenised with phosphate buffer 50 mM, pH 6.5. The homogenate was centrifuged at 6000× *g* for 25 min, and H_2_O_2_ concentration was determined using 0.1% titanium chloride in 20% (*v/v*) H_2_SO_4_. The amount of H_2_O_2_, detected spectrophotometrically (410 nm) and expressed as μmol g^−1^FW, was calculated by referring to a standard curve. Lipid peroxidation in roots and leaves was estimated by determining the amount of thiobarbituric acid reactive substances (TBARS) according to Spanò et al. [62]. Samples were mixed with the TBA reagent (10% *w/v* trichloroacetic acid + 0.25% *w/v* thiobarbituric acid), heated (95 °C for 30 min), cooled for 15 min, and centrifuged at 2000× *g* for 15 min. The concentration of TBARS was expressed as nmol g^−1^FW and calculated as specific absorbance at 532 nm by subtracting the nonspecific absorbance at 600 nm. 

### 3.8. Histochemical Analyses

Hydrogen peroxide and lipid peroxidation levels were visualized in situ using fluorescent probes specific for hydrogen peroxide and lipid peroxidation (Life Technologies, Carlsbad, CA, USA). Five roots of comparable developmental level, randomly selected from control and treated plants, were excised. Amplex UltraRed Reagent was applied for in situ detection of H_2_O_2_ following manufacturer’s instructions and the protocol reported in Giorgetti et al. [63]. Briefly, after staining, slices were mounted in glycerol and observed with fluorescence microscope (568ex/681em nm). BODIPY 581/591 C11 was used as a free radical sensor to visualize lipid peroxidation. The slices were incubated in 10 mM BODIPY in PBS 0.1 M, pH 7.4, for 30 min at room temperature in the dark and then washed three times in the same buffer. Microscope evaluation was performed, acquiring simultaneously the green (485ex/510em nm) and the red fluorescence (581ex/591em nm) signals and merging the two images.

### 3.9. Statistical Analysis

The design of the experiment was factorial, with two factors: Zn treatment with five levels (C, NPs, B, SPNPs, and SPB) and salt with two levels (addition of salt to the growth substrate and no salt supplementation). Data were analysed (statistic software package Past3) by a two-way ANOVA after checking for normality of distribution (Shapiro–Wilk test) and homogeneity of variances (Levene test). When required (*Fv/Fm*), they were log-transformed before ANOVA. When ANOVA highlighted significant differences, Tukey’s post hoc test was performed. The level of significance was *p* < 0.05.

## 4. Conclusions

ZnO treatment generally increased the concentration of zinc in wheat seedlings in a size- and mode-of-application-dependent manner, with severe alterations in root morphology. An increase in oxidative stress and damage was also recorded depending on the organ and on the size and mode of application of particles. Despite a more favourable ionic balance under saline conditions in ZnO-primed seedlings, growth was significantly inhibited and both decrease and increase in oxidative stress parameters were recorded. In conclusion, a negative effect was often associated with ZnO treatment and there was a large variability in the effects of priming on seedling salt response, both positive and negative depending on the organ and on the size or mode of amendment of ZnO. The presence of a cumulative negative effect of priming and salt stress sometimes recorded, in particular, call for caution in the use of ZnO in the protection from saline stress, particularly with regard to NPs which, due to their small size, can be better absorbed and translocated within plants in comparison with the bulk counterpart.

## Figures and Tables

**Table 1:** Na^+^, K^+^, and Cl^−^ concentration (mg kg^−1^) in root and leaf of *T. turgidum* L. ssp. *durum* cv. Cappelli without priming and salt treatment (C), with salt treatment without priming (NaCl), with ZnO treatment in the root zone (nanoparticles, NPs or bulk form, B), with ZnO priming in the root zone with salt treatment (NPs + NaCl or B + NaCl), with ZnO treatment as a leaf spray (SPNPs or SPB), and with ZnO priming as a leaf spray with salt treatment (SPNPs + NaCl or SPB + NaCl).

	Na^+^ in Root (mg kg^−1^)	K^+^ in Root (mg kg^−1^)	Cl^−^ in Root (mg kg^−1^)	K^+^/Na^+^ in Root	Na^+^ in Leaf (mg kg^−1^)	K^+^ in Leaf (mg kg^−1^)	Cl^−^ in Leaf (mg kg^−1^)	K^+^/Na^+^ in Leaf
**C**	189.78 ± 2.61 g	462.78 ± 41.59 h	270.53 ± 9.12 i	2.44 ± 0.23 d	1795.0 ± 29.44 f	12,535.0 ± 21.79 f	2496.0 ± 49.13 g	6.99 ± 0.06 d
**NaCl**	20,911 ± 63.88 b	5640 ± 119.1 g	30,834 ± 311.8 b	0.27 ± 0.01 e	33,993 ± 529.8 b	16,135 ± 680.9 de	48,503 ± 514.9 c	0.48 ± 0.03 f
**NPs**	1673.6 ± 25.21 f	15,676 ± 186.0 a	2650.0 ± 72.57 fg	9.37 ± 0.25 a	1052.0 ± 31.19 f	18,680 ± 23.28 c	1577.0 ± 79.17 g	17.79 ± 0.51 b
**NPs + NaCl**	22,368 ± 76.56 a	9113.1 ± 321.10 c	33,669 ± 188.7 a	0.41 ± 0.01 e	28,066 ± 33.30 c	23,141 ± 145.5 b	45,895 ± 471.3 d	0.82 ± 0.01 f
**B**	1365.3 ± 32.51 fg	12,467 ± 204.0 b	1792.0 ± 15.50 h	9.14 ± 0.09 a	1654.0 ± 30.57 ef	35,890 ± 118.2 a	2493.0 ± 47.42 g	21.72 ± 0.41 a
**B + NaCl**	16,997 ± 421.8 d	7342.4 ± 37.51 e	11,064 ± 83.43 e	0.43 ± 0.01 e	36,781 ± 189.6 a	16,125 ± 48.85 de	53,108 ± 106.6 b	0.44 ± 0.00 f
**SPNPs**	2040.0 ± 15.85 f	6526.0 ± 53.98 f	3056.0 ± 27.42 f	3.20 ± 0.04 c	2871.0 ± 8.82 e	14,461 ± 722.3 e	4439.0 ± 76.02 f	5.04 ± 0.25 e
**SPNPs + NaCl**	18,360 ± 455.0 c	6754.6 ± 89.38 ef	24,761 ± 121.6 c	0.37 ± 0.01 e	35,883 ± 107.0 a	16,607 ± 369.6 d	56,495 ± 692.5 a	0.46 ± 0.01 f
**SPB**	1879.4 ± 27.61 f	7680.4 ± 135.0 d	2528.0 ± 18.61 gh	4.09 ± 0.09 b	1607.0 ± 31.55 ef	24,992 ± 68.41 b	2064.0 ± 27.18 g	15.56 ± 0.30 c
**SPB + NaCl**	11,367 ± 298.0 e	9887.0 ± 10.05 c	16,403 ± 187.5 d	0.87 ± 0.02 e	23,792 ± 495.6 d	24,865 ± 335.4 b	38,645 ± 524.1 e	1.05 ± 0.01 f

Values are means of at least three replicates ± SE. When ANOVA highlighted significant differences, Tukey’s post hoc test was performed, and different letters indicate significantly different mean values at *p* < 0.05.

**Table 2:** Leaf number, pigment concentration, and maximum PSII quantum yield (*Fv/Fm*) in leaves of *T. turgidum* L. ssp. *durum* cv. Cappelli without priming and salt treatment (C), with salt treatment without priming (NaCl), with ZnO treatment in the root zone (nanoparticles, NPs or bulk form, B), with ZnO priming in the root zone with salt treatment (NPs + NaCl or B + NaCl), with ZnO treatment as a leaf spray (SPNPs or SPB), and with ZnO priming as a leaf spray with salt treatment (SPNPs + NaCl or SPB + NaCl).

	Leaf Number	Total Chlorophyll (mg g^−1^FW)	Carotenoids (mg g^−1^FW)	Chla/Chlb	*Fv/Fm*
**C**	2.80 ± 0.09 a	1.21 ± 0.07	0.21 ± 0.01	3.05 ± 0.02 a	0.79 ± 0.00
**NaCl**	2.10 ± 0.07 b	1.29 ± 0.08	0.22 ± 0.01	2.94 ± 0.10 ab	0.80 ± 0.00
**NPs**	2.95 ± 0.05 a	1.35 ± 0.04	0.19 ± 0.00	2.58 ± 0.05 bc	0.80 ± 0.00
**NPs + NaCl**	2.30 ± 0.10 b	1.28 ± 0.07	0.17 ± 0.01	2.49 ± 0.06 c	0.79 ± 0.01
**B**	2.70 ± 0.10 a	1.36 ± 0.11	0.20 ± 0.02	2.74 ± 0.03 bc	0.79 ± 0.01
**B + NaCl**	2.20 ± 0.09 b	1.44 ± 0.15	0.19 ± 0.02	2.58 ± 0.02 bc	0.78 ± 0.01
**SPNPs**	2.80 ± 0.09 a	1.39 ± 0.07	0.19 ± 0.01	2.65 ± 0.04 bc	0.79 ± 0.01
**SPNPs + NaCl**	1.95 ± 0.05 b	1.26 ± 0.10	0.17 ± 0.02	2.44 ± 0.14 c	0.79 ± 0.00
**SPB**	2.25 ± 0.10 b	1.45 ± 0.17	0.21 ± 0.03	2.53 ± 0.07 bc	0.76 ± 0.01
**SPB + NaCl**	2.10 ± 0.07 b	1.32 ± 0.08	0.19 ± 0.01	2.66 ± 0.08 bc	0.79 ± 0.01

Values are means of at least three replicates ± SE. When ANOVA highlighted significant differences, Tukey’s post hoc test was performed, and different letters indicate significantly different mean values at *p* < 0.05.

## Supplementary Materials

The following are available online at https://www.mdpi.com/2223-7747/9/11/1514/s1, Figure S1: Calcium concentration; Figure S2: experimental design; Table S1: F values from two-way ANOVA on root material; Table S2: F values from two-way ANOVA on leaf material.
