# Supplementary material for: Effect of Zinc Priming on Salt Response of Wheat Seedlings: Relieving or Worsening?
Source: Plants (Basel). 2020 Nov 8;9(11):1514. doi: 10.3390/plants9111514 (PMC7695260; doi:10.3390/plants9111514)
Supplement: Supplementary file 1 [file plants-09-01514-s001.zip › Table 1S.pdf]

Table 1S. F values from two-way ANOVA on root material

Zinc concentration

	df	F-value	*p*-value
Zn treatment	4	559.8	<0.001
Salt	1	41.67	<0.001
Zn treatment*salt	4	12.74	<0.001
Error	20		
Corrected total	29		

Na^+ concentration

	df	F-value	*p*-value
Zn treatment	4	165.5	<0.001
Salt	1	14118.00	<0.001
Zn treatment*salt	4	219.4	<0.001
Error	20		
Corrected total	29		

K^+ concentration

	df	F-value	*p*-value
Zn treatment	4	1102.00	<0.001
Salt	1	73.52	<0.001
Zn treatment*salt	4	541.00	<0.001
Error	20		
Corrected total	29		

Cl^- concentration

	df	F-value	*p*-value
Zn treatment	4	2270.00	<0.001
Salt	1	57660.00	<0.001
Zn treatment*salt	4	2419.00	<0.001
Error	20		
Corrected total	29		

K^+/Na^+ ratio

	df	F-value	*p*-value
Zn treatment	4	414.70	<0.001
Salt	1	4966.00	<0.001
Zn treatment*salt	4	418.30	<0.001
Error	20		
Corrected total	29		

Ca^{2+}

	df	F-value	*p*-value
Zn treatment	4	117.70	<0.001

Salt	1	0.70	0.4122
Zn treatment*salt	4	206.80	<0.001
Error	20		
Corrected total	29		

Root length

	df	F-value	*p*-value
Zn treatment	4	96.69	<0.001
Salt	1	33.70	<0.001
Zn treatment*salt	4	48.71	<0.001
Error	191		
Corrected total	200		

Relative water content

	df	F-value	*p*-value
Zn treatment	4	6.91	0.0012
Salt	1	0.13	0.7223
Zn treatment*salt	4	5.95	0.0025
Error	191		
Corrected total	200		

Hydrogen peroxide concentration

	df	F-value	*p*-value
Zn treatment	4	355.50	<0.001
Salt	1	9.08	0.005
Zn treatment*salt	4	71.37	<0.001
Error	30		
Corrected total	39		

TBARS concentration

	df	F-value	*p*-value
Zn treatment	4	42.79	<0.001
Salt	1	23.75	<0.001
Zn treatment*salt	4	104.10	<0.001
Error	30		
Corrected total	39		
