# Supplementary material for: Effect of Zinc Priming on Salt Response of Wheat Seedlings: Relieving or Worsening?
Source: Plants (Basel). 2020 Nov 8;9(11):1514. doi: 10.3390/plants9111514 (PMC7695260; doi:10.3390/plants9111514)
Supplement: Supplementary file 1 [file plants-09-01514-s001.zip › Table S2.pdf]

Table 2S. F values from two-way ANOVA on leaf material

Zinc concentration

	df	F-value	*p*-value
Zn treatment	4	2991	<0.001
Salt	1	2073	<0.001
Zn treatment*salt	4	324.0	<0.001
Error	20		
Corrected total	29		

Na^+ concentration

	df	F-value	*p*-value
Zn treatment	4	307.0	<0.001
Salt	1	38675	<0.001
Zn treatment*salt	4	238.1	<0.001
Error	20		
Corrected total	29		

K^+ concentration

	df	F-value	*p*-value
Zn treatment	4	440.8	<0.001
Salt	1	73.41	<0.001
Zn treatment*salt	4	404.4	<0.001
Error	20		
Corrected total	29		

Cl^- concentration

	df	F-value	*p*-value
Zn treatment	4	234.3	<0.001
Salt	1	41489	<0.001
Zn treatment*salt	4	147.1	<0.001
Error	20		
Corrected total	29		

K^+/Na^+ ratio

	df	F-value	*p*-value
Zn treatment	4	442.9	<0.001
Salt	1	6882	<0.001
Zn treatment*salt	4	420.9	<0.001
Error	20		
Corrected total	29		

Leaf length

	df	F-value	*p*-value
Zn treatment	4	12.80	<0.001
Salt	1	71.04	<0.001
Zn treatment*salt	4	0.8572	0.4908
Error	191		
Corrected total	200		

Leaf number

	df	F-value	*p*-value
Zn treatment	4	7.373	<0.001
Salt	1	113.3	<0.001
Zn treatment*salt	4	4.933	<0.001
Error	191		
Corrected total	200		

Relative water content

	df	F-value	*p*-value
Zn treatment	4	1.050	0.4064
Salt	1	127.9	<0.001
Zn treatment*salt	4	7.037	0.001
Error	20		
Corrected total	29		

Plants FW

	df	F-value	*p*-value
Zn treatment	4	2.474	0.0774
Salt	1	15.50	<0.001
Zn treatment*salt	4	1.214	0.3360
Error	191		
Corrected total	200		

Hydrogen peroxide concentration

	df	F-value	*p*-value
Zn treatment	4	1332	<0.001
Salt	1	4.113	0.0515
Zn treatment*salt	4	1134	<0.001
Error	30		
Corrected total	39		

TBARS concentration

	df	F-value	*p*-value
Zn treatment	4	121.1	<0.001
Salt	1	52.60	<0.001
Zn treatment*salt	4	140.1	<0.001
Error	30		
Corrected total	39		

Total chlorophyll

	df	F-value	*p*-value
Zn treatment	4	0.6534	0.6312
Salt	1	0.2816	0.6015
Zn treatment*salt	4	0.5232	0.7198
Error	20		
Corrected total	29		

Carotenoids

	df	F-value	*p*-value
Zn treatment	4	1.690	0.1918
Salt	1	1.341	0.2605
Zn treatment*salt	4	0.2753	0.8905
Error	20		
Corrected total	29		

Chla/chlb ratio

	df	F-value	*p*-value
Zn treatment	4	14.19	<0.001
Salt	1	3.934	0.0612
Zn treatment*salt	4	1.693	0.1911
Error	20		
Corrected total	29		

Fv/Fm

	df	F-value	*p*-value
Zn treatment	4	2.956	0.0623
Salt	1	2.329	0.1324
Zn treatment*salt	4	2.294	0.0701
Error	58		
Corrected total	67		
