# Supplementary figures and images for: Effect of Zinc Priming on Salt Response of Wheat Seedlings: Relieving or Worsening?
Source: Plants (Basel). 2020 Nov 8;9(11):1514. doi: 10.3390/plants9111514 (PMC7695260; doi:10.3390/plants9111514)

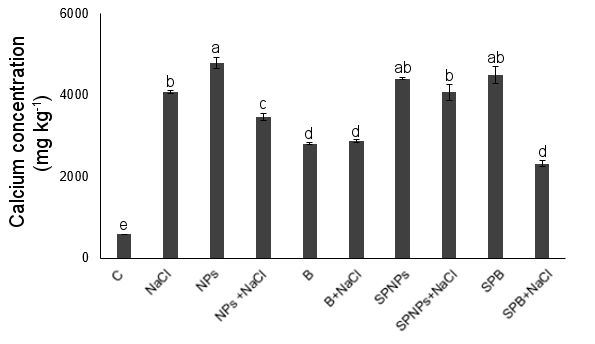

Supplement: Supplementary file 1 [file plants-09-01514-s001.zip › Figure 1S.tif]

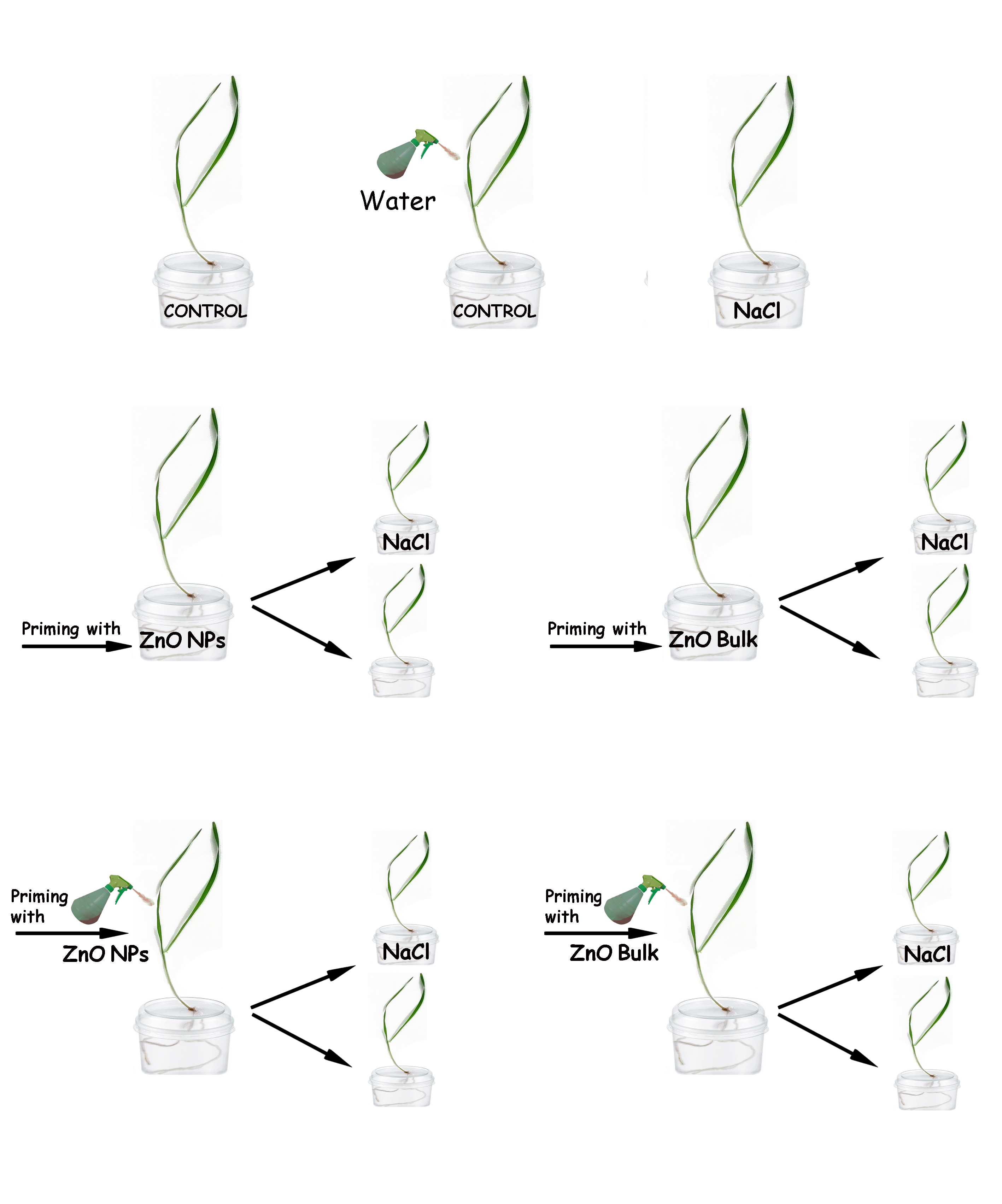

Supplement: Supplementary file 1 [file plants-09-01514-s001.zip › Figure 2S.tif]
